# The Effect of Surface Processing on the Shear Strength of Cobalt-Chromium Dental Alloy and Ceramics

**DOI:** 10.3390/ma15092987

**Published:** 2022-04-20

**Authors:** Liaisan Saleeva, Ramil Kashapov, Farid Shakirzyanov, Eduard Kuznetsov, Lenar Kashapov, Viktoriya Smirnova, Nail Kashapov, Gulshat Saleeva, Oskar Sachenkov, Rinat Saleev

**Affiliations:** 1 Kazan State Medical University, 420012 Kazan, Russia; saleeva.100mat@yandex.ru (L.S.); my@ekuznetsov.ru (E.K.); gulshat.saleeva@kazangmu.ru (G.S.); rinat.saleev@kazangmu.ru (R.S.); 2 Joint Institute for High Temperatures of the Russian Academy of Science, 125412 Moscow, Russia; kashramiln@gmail.com (R.K.); lenkashapov@kpfu.ru (L.K.); nail.kashapov@kpfu.ru (N.K.); 3 Institute of Engineering, Kazan Federal University, 420008 Kazan, Russia; 4 Kazan State University of Architecture and Engineering, 420043 Kazan, Russia; faritbox@mail.ru; 5 N.I. Lobachevsky Institute of Mathematics, Kazan Federal University, 420008 Kazan, Russia; yaikovavictoriya@mail.ru

**Keywords:** dental prosthesis, porcelain fused to metal, metal-ceramic, adhesion, profilometry, plasma-electrolyte processing, shear strength

## Abstract

Porcelain fused to metal is widespread dental prosthetic restoration. The survival rate of metal-ceramic restorations depends not only on the qualifications of dentists, dental technicians but also on the adhesive strength of ceramics to a metal frame. The goal of the research is to determine the optimal parameters of the surface machining of the metal frame to increase the adhesion of metal to ceramics. Adhesion of cobalt-chromium alloy and ceramics was investigated. A profilometer and a scanning electron microscope were used to analyze the morphology. To estimate the adhesion the shear strength was measured by the method based on ASTM D1002-10. A method of surface microrelief formation of metal samples by plasma-electrolyte treatment has been developed. Regimes for plasma-electrolyte surface treatment were investigated according to current-voltage characteristics and a surface roughness parameter. The samples were subjected to different surface machining techniques such as polishing, milling, sandblasting (so-called traditional methods), and plasma-electrolyte processing. Morphology of the surface for all samples was studied and the difference in microrelief was shown. The roughness and adhesive strength were measured for samples either. As a result, the mode for plasma- electrolytic surface treatment under which the adhesive strength was increased up to 183% (compared with the traditional methods) was found.

## 1. Introduction

The modern development of dental science and practice makes it possible to maintain the dental health of the population at a high level. Despite effective preventive measures, new technologies of treatment and dental prosthetics, dental morbidity remains at a high level worldwide [1,2]. In most cases, dental rehabilitation requires the use of dentures to restore the integrity of the dentition [3,4,5,6].

One of the most common and traditionally used dental prosthetic restorations is non-removable fixed dentures, in particular porcelain fused to metal (PFM) dentures. Many years of experience in the use of these prostheses shows their effectiveness, high survival over time and improves the aesthetics and quality of life of patients [7,8,9,10]. Despite this, in clinical practice there are often failures and complications associated with both errors in complex clinical planning and violations of laboratory stages during their manufacture.

There are enough publications in medical literature devoted to the issues of preserving the vitality of teeth selected as supports for metal-ceramic crowns, decementation of prostheses, inflammatory phenomena of the dentoalveolar papillae and others [11,12,13]. All of these complications have a human factor (dentist—dental technician) and can be caused by the individual characteristics of the patient. Also the manufacturing technologies of dentures are not quite perfect and require constant improvement, the development of new high-quality and biologically inert materials.

As materials used in the manufacture of prostheses is porcelain which is applied to the metal surface. Glass ceramics are obtained by melting glass using the method of directed crystallization. It consists of silicon oxide, commonly known as quartz (SiO_2_) with negligible aluminum content. The basic principle of obtaining a solid ceramic material is its molding when the molten glass cools. At the same time, during its sequential heating, a controlled crystallization process occurs, as a result of which crystals appear and grow. The process of transformation from pure glass to partially crystalline is called “ceramization”.

Noble metal alloys are widely used in dentistry [14]. However, base-metal alloy is an economical alternative to expensive gold alloy [15]. Nickel-chromium (Ni-Cr) and cobalt-chromium (Co-Cr) alloys are most widely used when cost and rigidity are taken into account [16,17]. However, the immediate advantage of the Co-Cr alloy is comparable performance to other base metal alloys, but without an allergenic nickel component [18], while titanium has low quality of metal-ceramic bond [19]. In prosthetic dentistry special grades of stainless steels are used, the so-called alloy steels: for stamping AISI 321 or AISI 321, for casting 20Cr18N9S2. The composition of stainless steels includes: 72% iron, 0.12% carbon, 18% chromium, 9–10% nickel, 1% titanium, 2% silicon. Alloy steels contain a minimum amount of carbon (its increase leads to an increase in hardness and a decrease in ductility of steel) and an increased content of specially introduced elements that ensure the production of alloys with the desired properties. Chromium gives resistance to oxidation. Nickel is added to the alloy to increase ductility and viscosity. Titanium reduces brittleness and prevents intercrystalline corrosion of steel. Silicon is present only in injection-molded steel and improves its fluidity. Stainless steel has good malleability and poor casting qualities.

The main problem in the manufacture of PFM restorations is the problem of poor adhesion of the ceramic coating to the metal frame [20,21,22]. Increasing the adhesion of materials will significantly increase the service lifetime of dentures, reduce costs, which in turn will improve the quality of life of patients.

One of the criteria for good adhesion is the absence of impurities on the two surfaces that will be compared. With regard to ceramic coating, the issue is at a high stage of solution and the dental technician only needs to observe the technology of applying the mass to the metal frame by excluding the ingress of impurities from the ambient air of the dental laboratory, then with regard to the preliminary preparation of the metal frame itself, a number of issues arise that require more in-depth study [23,24]. In particular, this concerns the use of sandblasting technology of the metal frame at the stages of cleaning from the molding mass after casting, preliminary mechanical grinding with milling cutters and finishing with an abrasive sand with a size of 50 μm. Unfortunately, the last stage does not exclude the possibility of introducing abrasive sand particles into the metal frame, which in the future may cause poor local adhesion and cause chipping of the ceramic veneering.

In addition, the shape, sharpness of the peaks and cavities of the microrelief, size, depth of surface defects and internal defects determine the strength of the material [25,26]. A review of the literature has shown that common clinical problems with ceramic materials are chips, marginal fractures and fractures of the main mass [27,28]. Volumetric fractures are still one of the main causes of failures in the use of PFM prostheses, but literary sources also suggest the long-term survival of various ceramic prostheses [29,30,31].

Due to the presence of surface roughness, developed cracks may not spread randomly, but occur at points with a higher stress concentration. The theory that crack nucleation begins at points of stress concentration caused by surface roughness was proposed by Mecholsky et al. [32], who loaded samples with grooves and furrows both perpendicular and parallel to the direction of loading.

Separately, a number of works devoted to the study of the processing of mating surfaces can be noted. Thus, Imbriglio et al. [33] investigated aluminum oxide and silicon carbide. A negative correlation was shown between the reduced powder size and adhesion. At the same time, the adhesion for silicon carbide did not statistically depend on the reduced powder size, and, as a consequence, roughness (the range of roughness 0.2–0.4 µm was studied).

Budhe et al. [34] investigated the adhesion to shear strength between aluminum and wooden samples during various surface processing. Thus, for aluminum samples, the maximum adhesion was achieved with a roughness of about 2 μm, but with an increase or decrease in roughness, the amount of adhesion decreased. The relationship between shear strength and surface roughness for wooden samples was nonlinear, the maximum adhesion was achieved at a roughness of about 1.5 μm, and then with a decrease in roughness, adhesion decreased. Similar results for shear strength of aluminum and steel were obtained by Ghumatkar et al. [35]. Murat et al. [36] investigated the pull-off adhesion testing for a medium-density fiberboard lined with polyvinyl chloride, and a nonlinear relationship between the amount of adhesion and roughness was also shown.

The purpose of the study is to increase the adhesion of cobalt-chromium alloy and ceramics by surface machining technology. Regimes for plasma-electrolyte treatment were investigated to find optimal surface microgeometry to increase the adhesion.

## 2. Materials and Methods

### 2.1. Study Protocol

The contact surfaces of the samples were subjected to different surface machining techniques such as polishing, milling, sandblasting (so-called traditional methods), and plasma-electrolyte processing. The traditional method of surface machining was carried out using a milling cutter NTI 060. 14.2 mm (NTI, Alfeld, Germany), a sandblasting machine (Bego Easyblast, Bremen, Germany) using sand (Renfert Cobra, Hilzingen, Germany) with particles size 125, 90, 50 μm at 0.25 MPa air-abrasion pressure approximately 10 mm from the sample surface during 60 s.

After surfacing, the samples were labeled. Then morphological and elemental analyses were carried out. After that samples with ceramic layers were manufactured. Shear strength was determined for joint samples.

### 2.2. Manufacturing of Samples and Surface Machining

**Figure 1:**
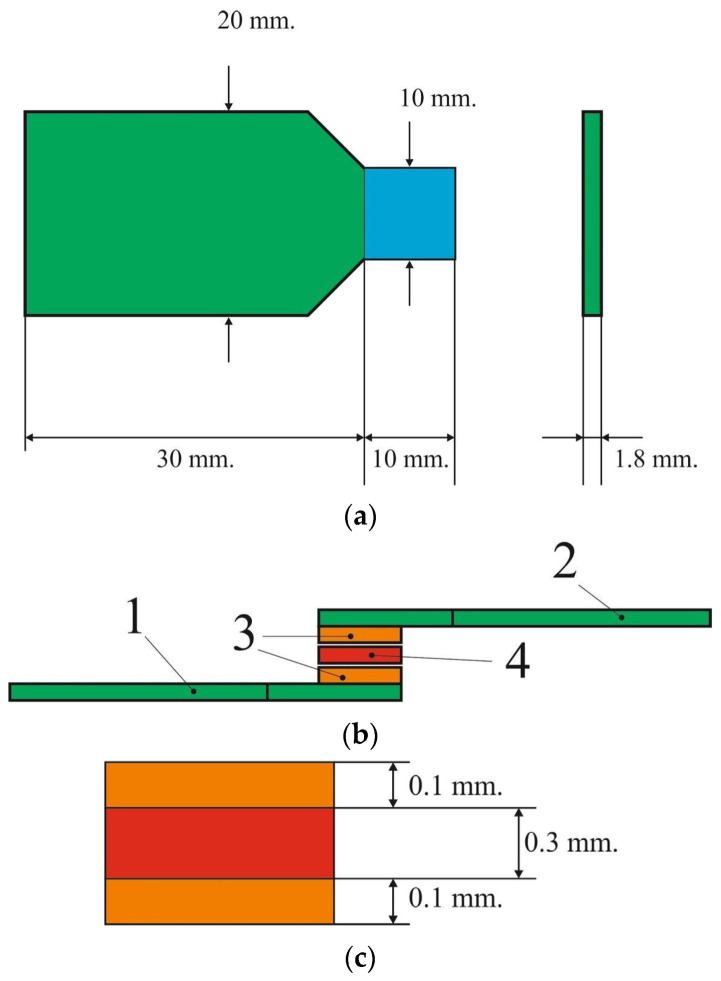
Sketch of the sample (**a**)—the sample, contacts surface is highlighted by blue; (**b**) the scheme of joint the samples: 1, 2—samples, 3—opaque layer, 4—ceramic layer; (**c**) the scheme of the joint layer: opaque and ceramic layers thickness.

The wax patterns were fabricated using basic wax “Stoma” (Kharkiv Oblast, Ukraine). The cobalt chromium metal alloy “I-bond NF” (Interdent d.o.o., Slovenia) (Co 63%, Cr 24%, W 8%, Mo 3%, So 1%) was used to cast a metal framework using Bego Fornax T (BEGO Bremer Goldschlägerei Wilh. Herbst GmbH & Co. KG, Germany). The geometry of samples is shown in Figure 1a.

Then the mating/contact surfaces were processed (the blue area in Figure 1a). The metal frame was veneered with IPS Inline ceramic mass (Ivoclar vivadent, Germany), firing was carried out in a Programat E5000 furnace (Ivoclar vivadent, Germany) by following instructions of the manufacturer [37].

At the first stage of manufacturing PFM restoration, an opaque layer was applied to two separate samples (see Figure 1), which was then sintered in an oven under firing mode I in Table 1. After firing, the samples were cooled and treated with an Emmevi (Hansgrohe-Axor, Germany) steam jet in a pressure mode of 0.6 MPa. The second layer was re-applied with an opaque layer and the same treatment was carried out. The next step was to apply a layer of porcelain to connect the two samples according to the scheme shown in Figure 1b, and baked in a connected form in the oven in mode II (Table 1). The thickness of the opaque layer and ceramics are shown in Figure 1c. Two connected samples were cooled after sintering and subjected to steam jet treatment. At the last stage, a layer of dentin was applied along the edge of the junction of the two samples and baked in mode III (Table 1). After cooling, the sample was cleaned using a steam jet machine.

### 2.3. The Technique of Plasma-Electrolyte Formation of the Microrelief of the Surface of Cobalt-Chromium Alloys

The method of surface microrelief formation of metal samples by plasma-electrolyte treatment consists of the use of gas discharges with liquid electrodes. The treatment process occurs as a result of the gas discharge combustion on the surface of a metal electrode dipped in an electrolyte solution. Depending on the polarity of the electrode and the type of material, various processes can occur: surface polishing, oxide ceramic coating formation, changes in surface roughness, application of metallic nanostructured coatings etc. To achieve our goal—control changes in roughness—it is necessary to use the cathode polarity of the active processed electrode.

**Figure 2:**
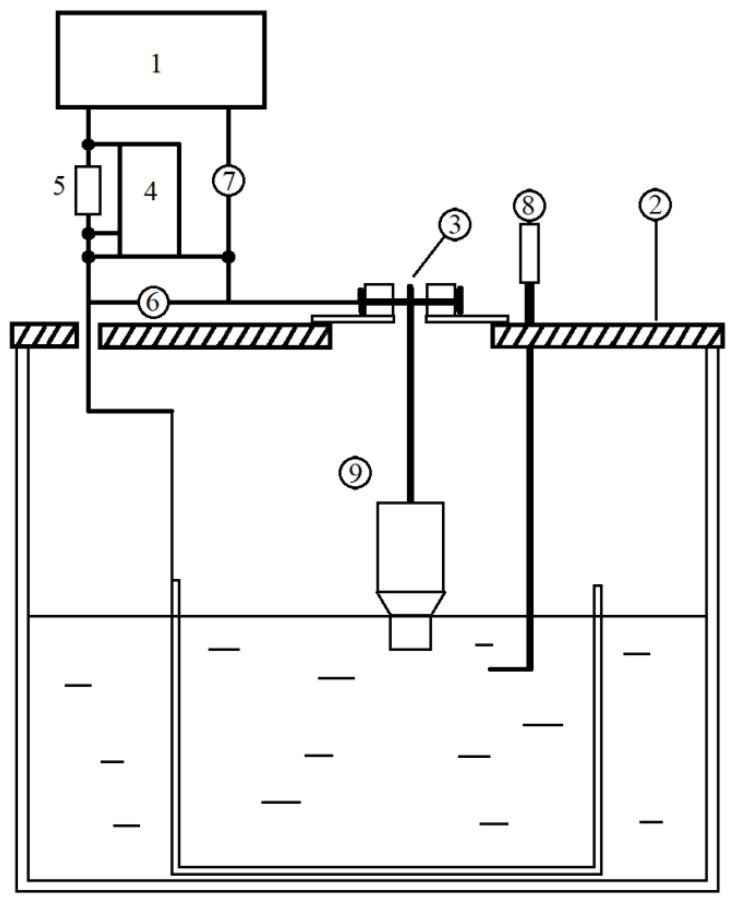
Scheme of the experimental installation of plasma-electrolyte formation of the microrelief of the surface: 1—electric power supply, 2—an electrolytic bath, 3—an electrode system, 4—an oscilloscope, 5—an additional resistance, 6—a voltmeter, 7—an ammeter, 8—a thermocouple and 9—a fixed sample.

The flat plate of the sample (9 in Figure 2) was fixed and immersed to a certain depth by the electrode system (3 in Figure 2). The work used a DC power supply (1 in Figure 2) with a continuously adjustable voltage, consisting of a diode bridge (diodes SD 246) and a laboratory autotransformer 1 M with a voltage range from 1 to 400 V (depending on the experimental conditions, a smoothing capacitor filter (C = 1560 μF) is connected to the power supply). Additional resistance (5 in Figure 2) also was added. The voltage and discharge current were measured using two digital universal measuring devices APPA 305 (6 in Figure 2) and APPA 109N (7 in Figure 2), the relative measurement error is 0.8%. An oscilloscope FLUKE scopemeter 190-062 (4 in Figure 2) was used to monitor the system. Electrolytic bath (2 in Figure 2) was filled with electrolyte—sodium chloride aqueous solution of concentration: 1%, 3% and 5% by weight. For each concentration of solution was carried out removal of the current-voltage characteristics of the plasma-electrolyte treatment. Temperature measurements were carried by thermocouple (8 in Figure 2).

The main parameters affecting the treatment process are the magnitude and shape of the applied voltage, the current strength of the discharge circuit and the temperature of the electrolyte. In the experiments, a smoothed voltage form obtained by using a capacitive filter was used, and by adjusting it, we changed the processing mode. The use of an active processed cathode polarity electrode leads to local melting of its surface under the action of randomly occurring single microdischarges. Depending on the discharge power and the temperature of the electrode itself, the formation of various microholes is observed, which in turn collectively form the overall surface roughness. The formation of microholes occurs as a result of melting of the surface and partial release of the electrode material into the electrolyte. Different treatment regimen forms different surface roughness. So a large number of samples for the case of plasma-electrolyte treatment is explained by the variety of choice of combustion regimes for discharges with liquid electrodes.

### 2.4. Morphological and Elemental Analysis

The study of the surface morphology was carried out with a scanning electron microscope (SEM) “EVO 50 XVP” (Carl Zeiss, Jena, Germany) with a probe microanalysis system “INCA Energy—350” (Oxford Instruments, Abingdon, UK). Roughness parameters were determined using a TR-200 profilometer (TIME GROUP Inc., Beijing, China). According to the curves of the surface profilograms, the values of the roughness parameters were calculated: Ra is the mean deviation of the profile; Rq is the mean square deviation of the profile. As a result of the research, it was found that the total error is less than 5%.

### 2.5. Apparent Shear Strength Determining

Researchers divide methods for determining adhesion into two groups: the pull-off strength measurement of adhesion and the apparent shear strength of single-lap-joint adhesively bonded specimens. Nowadays there are a number of standardized methods for their determination: ISO 4624:2002, ASTM C633-01, ASTM D7234-05, ASTM D1002-10, ASTM D4541, ISO 9693-1:2012. Mainly these standards were developed for paints and varnishes, for thermal spray coating materials, for rigid plastics, for metal to metal. For metal-ceramic materials, standardized methods have not been found during the study of this issue (except ISO 9693-1:2012). ISO 9693-1:2012 is focused on normal strength, but in the research the focus was on shear strength. It leads from the assumption that surface morphology critically influences shear [32]. Therefore, in this work, an approach to determine the apparent strength is based on the developed standards for adhesively bonded metal specimens. In this regard, for determining the apparent shear strength of the adhesive ceramic layer taken as a basis ASTM D1002-10 (Standard test method for apparent shear strength of single-lap-joint adhesively bonded metal specimens by tension loading) [38]. Similar approach was used in other researches [34,35,36].

**Figure 3:**
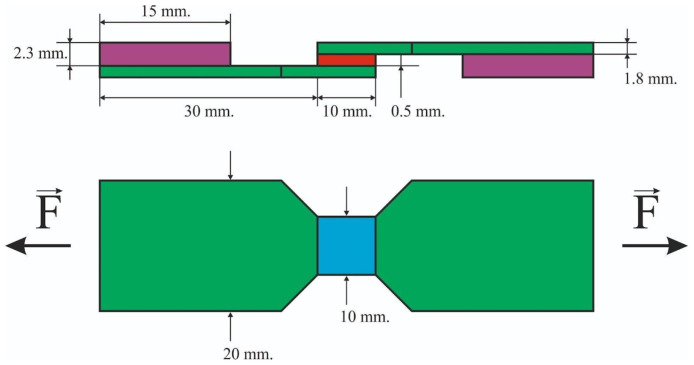
Sketch of the sample, dimensions and loading scheme: the ceramic layer is highlighted by red; the cobalt-chromium sample is highlighted by green; the centering plates highlighted by magenta; the contact surfaces highlighted by blue.

The samples were tested on a universal testing machine UTS 110M-100 (Ivanovo, Russia). The range of measured loads is 0.001–100 kN, load measurement error less than 1% from the statement, up to 1/100 from the value of the permissible load. The test method is to obtain the value of the tensile load of failure of two samples bonded together with ceramics. The forces tending to shift one half of the sample relative to the other showed in Figure 3, strain rate was equal to 1 mm/min. To decrease the bending stresses during the shear test, the thickness of the metal frames was equal to 1.8 mm. Shear strength can be calculated by the following equation:

(1) $$\tau =\frac{F}{A}$$

where *F* is the failure load (N), *A* is the area (mm^2^) of the contact surface (ceramic joint).

### 2.6. Statistical Processing of Experimental Data

Statistical analysis of the data was carried out using the MatLab software. The results are given in the following format: average ± half of the confidence interval (*p* < 0.05). Nonparametric measurements are given in the following format: median (Me) and interquartile range of 25–75 (Q1–Q2). A normal distribution check was carried out using the Jarque-Bera test. To compare groups two-sample *t*-test (*p* < 0.05) and Kolmogorov–Smirnov test were used. The interpolation of the data was carried out using the least-squares method.

## 3. Results

Samples were prepared according to the study protocol and divided by groups with the following marks: polishing (*n* = 3)—PL; milling (*n* = 3)—MC; abrasive blasting, with an abrasive size of 50 μm (*n* = 12), abrasive size 90 μm (*n* = 3), abrasive size 125 μm (*n* = 3)—AB50, AB90, AB125 respectively; plasma-electrolyte processing (*n* = 16)—PZ.

### 3.1. The Surface Microrelief Formation by Plasma-Electrolyte Treatment

During plasma-electrolyte treatment, the surface relief formation occurs due to the combustion of individual microdischarges that melt the surface. For local melting, it is necessary that at the ignition point of the gas discharge, the temperature of the cobalt-chromium alloy S&S Scheftner (Mainz, Germany) becomes higher than the solidus temperature of 1170 °C, preferably above the liquidus temperature of 1390 °C. The melting process is beginning when the solidus temperature is exceeded. A completely liquid metal can be obtained only at temperatures above 1390 °C. It is known that the casting temperature recommended by the manufacturer is 1490–1540 °C. At these temperatures the alloy has good fluidity and there is no burnout of alloying elements. Insufficient invested discharge power will not allow to achieve the required heating of the sample and lead to local melting with the formation of microholes. And a higher power can lead to overheating of the entire sample and its melting with loss of geometry, which is unacceptable. It should also be noted that the treatment proceeds more intensively on sharp and protruding surfaces, this is explained by the greatest intensity of the electric field in these places.

Thus, when processing samples, it is necessary to take into account their geometry, mass and properties of the alloy. With a sample thickness of a metal plate of 1.8 mm, surface melting occurred at a voltage of U = 220 V and a current strength of I = 12 A, and at lower voltage values, samples with a roughness parameter Ra from 0.77 to 2.51 μm were obtained.

At the initial moment of the treatment, the electrolyte is in contact with the surface of the metal sample. Depending on the magnitude of the applied voltage and the temperature of the electrolytic anode, one of the following processes will take place: only electrolysis; electrolysis eventually turning into the combustion of discharges on the surface of the sample; initial combustion of discharges without the stage of electrolysis. In the case of electrolysis, a linear dependence of the current strength on the voltage will be observed. According to the Lenz-Joule law, when an electric current passes through a metal electrode (cathode), the amount of heat released is directly proportional to the square of the current, the resistance of the electrode and the time during which the electric current flowed. An increase in voltage leads to an increase in current. This leads to an increase in the degree of cathode heating. On the cathode surface the process of boiling the electrolyte begins, in addition to the electrochemical release of gas bubbles. Combustion of discharges occurs in gas bubbles. The flow of the above processes is determined by the current-voltage characteristic (CVC) of the treatment, the behavior of which changes when the type of electrolyte, its concentration and temperature change. By changing the dependence of the current strength on the applied voltage, it is possible to determine the intensity of electrochemical reactions, vaporization near the surface of the metal electrode and combustion of single discharges.

**Figure 4:**
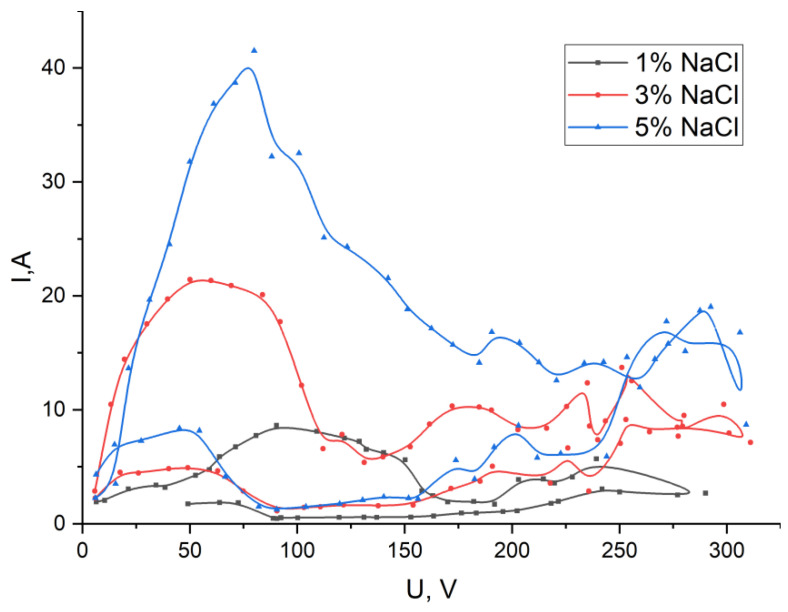
CVC of plasma-electrolyte treatment for NaCl solution: By grey line is 1%, red line is 3%, blue line is 5%.

Let us consider the CVC (Figure 4) of the plasma-electrolyte treatment of a cobalt-chromium alloy obtained using 1, 3 and 5% sodium chloride solutions. The CVC consists of two branches: the first is a gradual increase in voltage from 0 to 300 V, the second is a decrease in the applied voltage from 300 to 0 V.

For a 1% aqueous solution of sodium chloride, the combustion of single discharges with a pink-purple glow color occurs at a voltage of 120 V (grey line in Figure 4). When the applied voltage exceeds 100 V, local air-gas jets are observed departing from the cathode surface by 3–4 mm from the surface. On the CVC curve in this section, the beginning of a decrease in current strength is observed. A gradual increase in voltage leads to an increase in the number of single discharges. At a voltage of 177–181 V, local discharges of a different glow (yellow-orange) are additionally observed. At a voltage of 193 V, only yellow-orange glow discharges remain. The process of intensive heating of the electrode begins, it heats up to a temperature of 850–900 °C. At a given temperature of the electrode, a film boiling of the electrolyte will occur. Film boiling presupposes the presence of a stable vapor-air shell that covers the surface of the electrode. However combustion of discharges leads to a violation of the film boiling of the electrolyte and its splashing. The glow at the tip of the electrode turns white and corresponds to approximately a temperature of 1300 °C, in other areas of the surface it turns yellow and corresponds to a temperature of 1000 °C. Combustion of “yellow” discharges stops to prevail on the second branch of the CVC with a decrease in voltage to 216 V, mainly the combustion of electrical “pink-violet” discharges. Combustion of individual “yellow” discharges of low intensity occurs with a decrease in voltage to 163 V. When the voltage drops below 90 V, combustion stops and electrochemical reactions occur with boiling of the electrolyte near the electrode surface.

The use of a 3% aqueous solution of sodium chloride as an electrolyte changes the CVC curve while maintaining its outlines (red line in Figure 4). Namely, at 45 V—sound vibrations appear, at 60 V—the beginning of combustion of individual discharges on the cathode surface is observed. As the voltage increases, the number of discharges increases. The glow has a pink-purple color. At 100 V, larger yellow discharges appear. At 113 V, only yellow discharges burn. With increasing voltage, the intensity of radiation and acoustic vibrations increases. There are strong current fluctuations associated with intense splashing of the electrolyte near the cathode electrode. With a decrease in the applied voltage, the reverse transition occurs at 83 V. Local melting of the electrode surface is observed.

An increase in the concentration of sodium chloride in the electrolyte to 5% contributes to the displacement of the CVC to the region of lower voltages and an increase in the current strength (blue line in Figure 4). A voltage to 65 V increase leads to acoustic vibrations in the form of a crackle. At 67 V the combustion of individual discharges on the electrode surface begins. The color of the discharge radiation is yellow. At 88 V acoustic vibrations of a different frequency occur. With a decrease in voltage, the reverse transition from the combustion of discharges to the flow of only electrochemical reactions is observed at 63 V.

For all three CVCs with different concentrations of electrolyte, the presence of hysteresis is characteristic, with a reverse decrease in voltage, the current strength becomes significantly less. This is due to the heating of the electrolyte and the processed metal electrode. However, to a greater extent from the heating of the electrode, since it creates a film boiling that separates the electrolyte from the metal surface, and the absence of contact no longer allows electric current to be conducted due to electrochemical reactions. The current flow occurs only due to combustion of electrical discharges.

The obtained results of the CVC curves of plasma-electrolyte treatment indicate that a 3% solution of sodium chloride is the most optimal for use. With this sodium chloride content, there is no flow of high current densities for both 5% of the solution concentration and the risk of sample melting is reduced, and the use of 1% solution requires the use of higher voltage values to achieve the required energy supply. Based on the CVC curve for a 3% solution, the samples were processed to create roughness in the range from 120 to 200 V.

**Figure 5:**
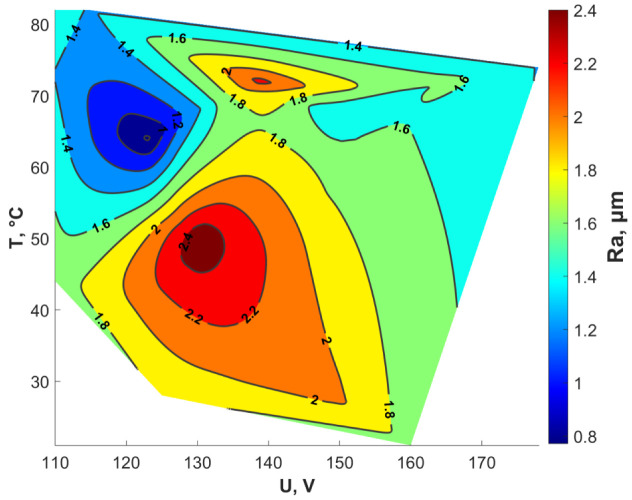
Roughness distribution depending on voltage and temperature of the electrode.

Varying plasma-electrolyte regimes generate different values of roughness. So, data of voltage, the temperature of the electrode, and the received roughness was interpolated by Delaunay triangulation (the natural neighbor interpolation method in Matlab). The obtained results are shown in Figure 5. The received interpolation allows selecting the plasma-electrolyte treatment mode to obtain a given roughness.

### 3.2. Analysis of the Microrelief of the Surface after Treatment

It should also be noted the gradient nature of the formation of the microrelief of the surface along the immersion of the sample in the electrolyte. The maximum size of the holes at the end of the sample, however, as they gradually move away from the edge, their size decreases. Using the example of changing the surface roughness parameters of the samples, it can be said that Ra in the longitudinal direction changed as follows: 2.734→2.064→1.728→1.375 μm.

**Figure 6:**
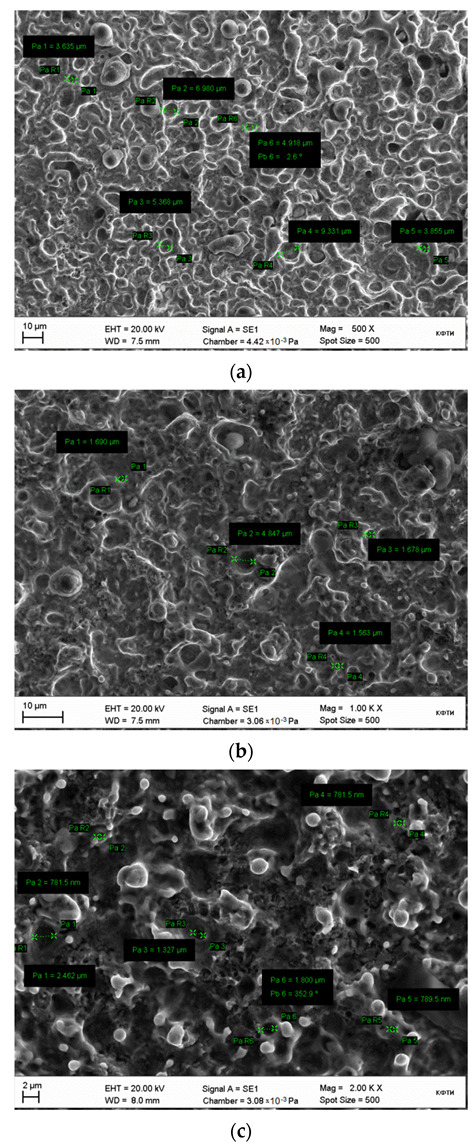
SEM images of the sample surface after treatment: (**a**)—the 1st research area with 500× magnification, (**b**)—the 2nd research area with 1000× magnification, (**c**)—the 3rd area of study of the sample with 2000× magnification.

Figure 6 shows pictures of the characteristic treated surface of the sample obtained by scanning electron microscopy. The images show that, depending on the depth of immersion of the electrode, the morphology of the surface changed. Conventionally, the surface can be divided into several sections, differing in morphology and size of microholes. The first region covers the end of the sample with a length of 2.5 mm, its morphology of the surface of the 1st region is very developed (Figure 6a). The diameter of microholes varies from ~3 μm to ~10 μm. Figure 6b shows the 2nd research area with 1000× magnification, which is 2.5 mm away from the edge of the sample, its surface morphology is developed. The diameter of microholes varies from ~1.5 μm to ~5 μm.

Figure 6c shows the 3rd area of study of the sample with 2000× magnification, which is removed from the edge of the sample by 5 mm, its morphology is very developed. The diameter of microholes varies from ~500 nm to ~2 μm.

It is established that the magnitude of the applied voltage strongly affects the nature of the plasma processes and heat generation. At insufficient voltages, the processing effect is not observed, and at higher voltages, melting of the electrode may occur. The most optimal voltage range is 160–190 V. Also an interesting fact is the strong heating of the electrode and its glow to red when using a new electrolyte at room temperature at the initial moment of gas discharge combustion, but after a certain time the effect of incandescence of the cathode passes, and it acquires a natural color. At the end of the process, if the electrode is removed from the electrolyte in a heated state, without contact with water and its natural cooling in air, then it is covered with a dark oxide film. If the electrode is cooled in the electrolyte, there is a transition from film boiling to contact boiling, with local near-electrode boiling of the electrolyte, and the formation of a black oxide film does not occur.

**Figure 7:**
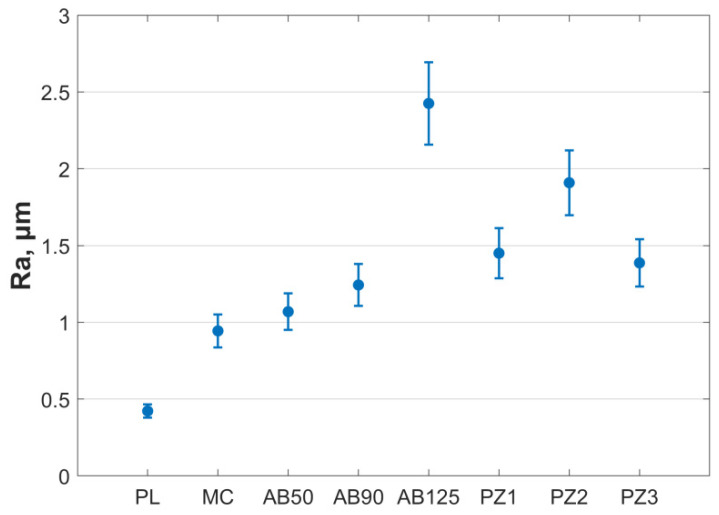
Surface roughness parameter distribution of samples, where bars represent mean (circle) and standard deviation (bars).

Sandblasting and new plasma-electrolyte treatments were compared. The surface roughness parameter Ra of cobalt-chromium samples after various processing methods presented in Figure 7. Grouping in ascending order of Ra value: PL, MC, AB50, AB90, AB125. The PZ group was divided into three groups according to Ra values: PZ1, PZ2, and PZ3. The values of Ra in groups are described in details in Section 3.3.

A comparison of the roughness parameters of standard surface treatment methods with plasma-electrolyte microrelief formation shows that this method is superior in its capabilities to sandblasting, milling and surface polishing. Thus, by adjusting the parameters of the processing process, it will be possible to obtain the required surface roughness.

A complex relationship exists between surface roughness and adhesion. As expected, when a larger particle size abrasive is used, the Ra value is seen to increase. However, the average roughness increases as the larger particles create higher peaks and deeper valleys in the surface profile [39]. Also in [39] it was shown that as the Ra value increases, the Rz and Rq values increase proportionally.

**Figure 8:**
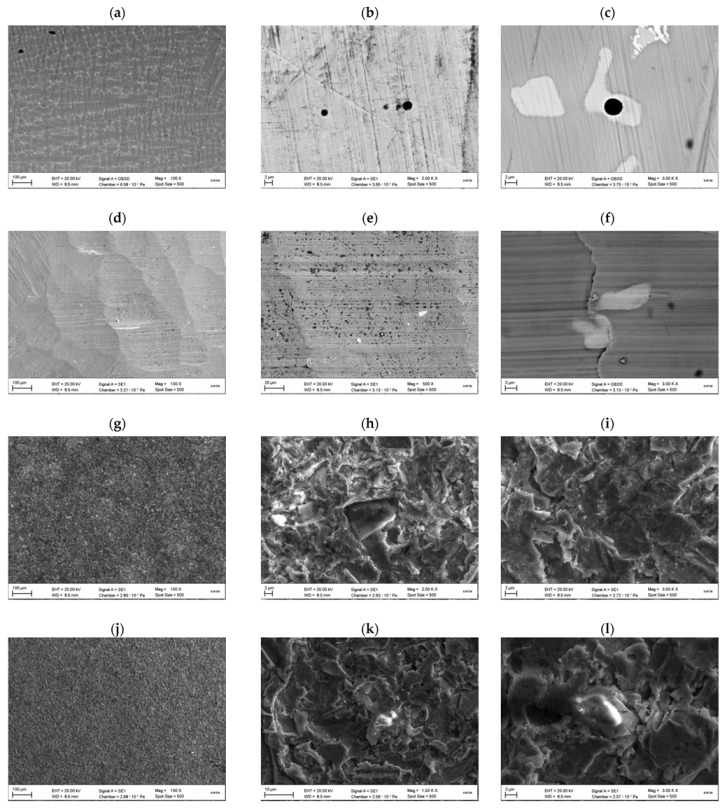

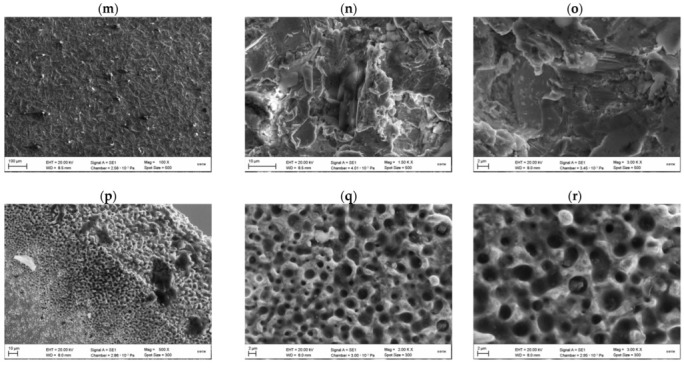
SEM images of the surface for various processing methods: (**a**–**c**)—surface after polishing (PL group); (**d**–**f**)—surface after milling (MC group); (**g**–**i**)—surface after sandblasting with an abrasive size of 50 μm (AB50 group); (**j**–**l**)—surface after sandblasting with an abrasive size of 90 microns (AB90 group); (**m**–**o**)—surface after sandblasting using with an abrasive size of 125 microns (AB125 group); (**p**–**r**)—surface after plasma-electrolyte treatment (PZ groups).

A comparative assessment of the surface morphology for various processing methods was carried out. SEM images of the surface for various processing methods presented in Figure 8. The surface after polishing is presented in Figure 8a–c. Irregularities in the form of cavities formed by the abrasive of the polishing tool are detected at 2000× and 3000× magnification. The surface after smearing the surface layer with a processing tool presented in Figure 8d–f. Surface after sandblasting using sand with a dispersion of 50 μm presented in Figure 8g–i. In these images can be observed the remains of sand particles pressed into the metal base. The morphology of the surface has many sharp protruding irregularities left as a result of the sliding of sand particles on the metal surface.

Figure 8j–l show images of the surface after sandblasting using sand with a dispersion of 90 μm. Figure 8m–o show images of the surface after sandblasting using sand with a dispersion of 125 μm. Sand particles are found on all the studied surfaces. The main difference is the size of the formed irregularities.

Figure 8p–r show images of the surface after plasma-electrolyte formation. A porous structure is observed, the protrusions have a spherical shape. The surface obtained by this method is fundamentally different in morphology from standard processing methods in the absence of sharp ledges, which later, when applying ceramics, can act as stress concentrators and lead to chipping of ceramics under cyclic loads.

### 3.3. Apparent Shear Strength

**Figure 9:**
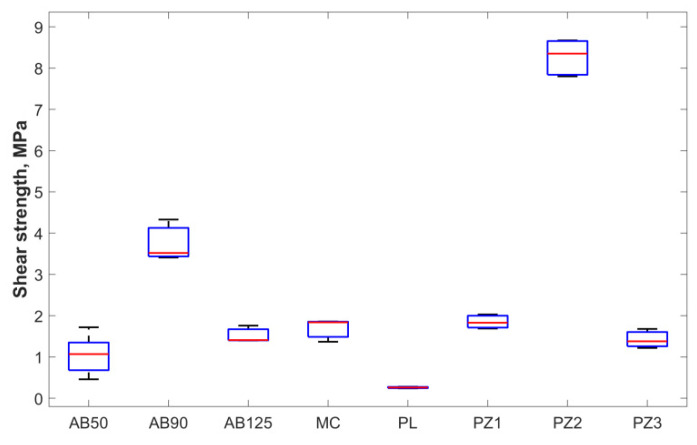
Shear strength distribution by groups, where are shown: the median (red line), the lower and upper quartiles (blue lines), and the minimum and maximum values (black lines) that are not outliers.

The distribution of shear strength by groups is presented in Figure 9. There was a significant decrease in adhesion strength for the PL group (*p* < 0.05). Shear strength in group AB90 was significantly higher than in group AB50 (*p* < 0.05) and insignificant higher than in group AB125. Groups AB50, AB125, and MC did not differ significantly. In plasma-electrolyte groups the shear strength of PZ2 was significantly higher than in PZ1 and PZ3. Ra and shear strength results in groups are presented in Table 2.

**Figure 10:**
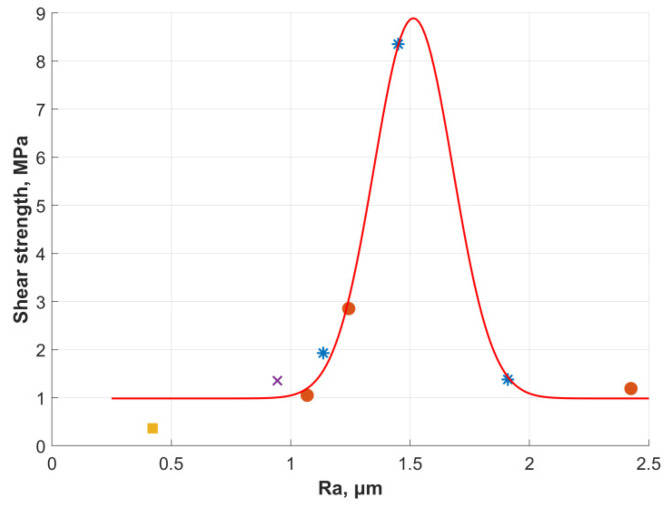
Distribution of average shear strength depending on Ra value, rectangle—PL, cross—MC, circle—AB (from left to right 50, 90, 125), stars—PZ (from left to right PZ1, PZ2, PZ3) and interpolation result—red line.

Thus, when considering the relationship between the roughness parameter Ra and the shear strength for the PZ group a nonlinear relationship was noted. Due to the nonlinear dependence of shear strength on Ra for the PZ group, their averaging over the full data set is incorrect. So the data from the PZ group were divided according to the Ra values into three groups (see Figure 7). For the PZ1 group from PZ roughness Ra value was equal to 1.136 ± 0.15 µm and the shear strength was 1.93 ± 0.10 MPa, for the PZ2 group Ra was 1.45 ± 0.16 µm and the shear strength was 8.35 ± 0.21 MPa, for the PZ3 group Ra was 1.91 ± 0.21 µm and shear strength was 1.38 ± 0.07 MPa. Figure 10 shows the average values of shear strength and Ra value for all groups.

Assuming the homogeneity of all data, thereby excluding the influence of the chemical component and microrelief, it is possible to construct an exponential interpolation (red line in Figure 10) of the form:

(2) $$\tau \left(Ra\right)=a+b\cdot \frac{1}{\sigma \sqrt{\pi }}Exp\left(\frac{{\left(Ra-\mu \right)}^{2}}{2\sigma^{2}}\right)$$

The parameters of interpolation (2) were as follows: *a* = 0.984 MPa, *b* = 3.271 MPa∙μm, *μ* = 1.514 μm, *σ* = 0.165 μm, error of interpolation was calculated as a squared norm of the residual (r^2^) and was equal to 0.7564.

Due to the form of interpolation, the curve reaches a plateau in intervals of Ra values up to 1 µm and over 2 µm. In a strict sense, this fact illustrates a bad interpolation, but values of shear strength in these intervals are too small and these intervals are of no practical interest. Interpolation describes the results obtained quite qualitatively in the range of Ra values from 1 µm and up to 2 µm. Summarizing, the received interpolation and previous interpolation of roughness distribution depending on voltage and temperature of the electrode (see Figure 5) it is possible to select the appropriate mode of plasma-electrolyte treatment to obtain the required shear strength.

## 4. Discussion

The developed method of microrelief formation by plasma-electrolyte treatment allows to obtain the required surface roughness of metal frame by changing the parameters of gas discharge combustion. The microrelief arises due to combustion of individual microdischarges that melt the surface and create roughness. Size of microholes can be changed by controlling microdischarge parameters. This method of processing allows to obtain a clean surface, devoid of foreign impurities that may occur during abrasive processing. This method gives an opportunity to achieve the same results on the same parameters of plasma-electrolyte treatment. Also plasma-electrolyte treatment leads to the formation of a microrelief with spherical shape hollows and protrusions that can reduce the local stress concentration [40,41,42].

The main application of the plasma-electrolyte treatment is realized for surface polishing [43], heat treatment of products [44] and the formation of functional coatings [45]. In the presented work, the combustion of gas discharges with liquid electrodes was used to form a microrelief or controlled change in surface roughness. This application of the plasma-electrolyte treatment is achieved due to the possibility of local melting of the surface by single microdischarges. It should be mentioned that the study of the influence of the plasma-electrolyte treatment on the surface of the CoCr alloy was carried out on flat samples. Actually a dental crown has a complex geometry with different wall thicknesses. In the case of uniform burning of individual discharges on the surface of the dental crown, uneven heating of different parts of the crown is expected. But since the process takes place in an aqueous electrolyte solution, overheating of the CoCr alloy doesn’t occur. However, there may be a change in the power of individual microdischarges depending on the geometry of the crown, which can lead to different roughness parameters, such as Ra. So, changes in roughness parameters for different geometries and types of metal frames of dental crowns should be investigated additionally.

For the proposed treatment method, as well as for classical methods, shear strength was measured. It was shown a nonlinear relationship between roughness parameters Ra and the shear strength value, such results correlate with other authors [34,35,36]. It was proposed to describe the obtained dependence by exponential interpolation. Such interpolation was received excluding the influence of the chemical component and microrelief over shear strength. Unlike plasma-electrolyte treatment, traditional methods (polishing, milling, and sandblasting) provide sharp geometry of hollows and protrusions.

As the result shear strength nonlinearly depends on the value of the roughness parameters. Similar trend of shear strength with respect to surface roughness was found by Budhe et al. [34], Ghumatkar et al. [35] and Sekercioglu et al. [46]. Thus, the maximum shear strength appears near 1.5 µm Ra value for aluminum AA6061 [34] and steel S235JR—EN 10,025 [46]. Ghumatkar showed that the maximum shear strength appears near 2 µm Ra value for aluminum AA6063 and steel AISI1045 [35]. Comparing the dependence between roughness and shear strength for AA6061 and AA6063 it can be noted that maximum value remains about 5 MPa. Meanwhile the roughness (in terms of Ra) was about 1.75 µm for AA6061 and 2.25 µm for AA6063. Similar results obtained for AISI1045 and S235JR—EN 10,025—maximum shear strength localized at Ra values equal to 1.8 µm and 1.5 µm respectively. The influence of chemical adhesion with the frame microrelief is observed indirectly. It reflects on the absolute value of adhesion during the shear strength. So, the fluctuation of Ra value for maximum shear strength can be explained by chemical adhesion and the number of roughness points in the experiments.

A shear adhesion study showed that with standard metal surface treatments, the dispersion of test results was not big and it was sufficient to carry out tests on 3 samples. In contrast, plasma treated samples gave a large dispersion; so 16 samples were carried out. Subsequently, it turned out that the dispersion of results is greatly affected by temperature and current intensity.

So, the certain shear strength can be reached according to the received interpolation between mode and roughness and between roughness and adhesion. This increasing the value of the shear strength by 183% compared to the traditional method. The obtained results allow us to estimate the localization of the maximum shear strength depending on the roughness. Despite it, the determination of shear strength magnitude according to roughness and surface machining is still open, since it is influenced by other characteristics of the microrelief, chemical adhesion either.

The proposed method obtained samples with a value of Ra equal to 1.45 ± 0.16 µm and shear strength equal to 8.35 ± 0.21 MPa. The shear strength exceeds the same indicator for sandblasting by almost two times.

## 5. Conclusions

The high prevalence of dental diseases and untimely dental treatment leads to the loss of natural teeth. Missing or damaged teeth could be replaced by dental prosthesis, including fixed metal-ceramic restorations. The functional duration of dental prosthesis depends on many factors and one of them is the preservation of the integrity of the dental prosthesis by itself. Despite using PFM restorations various complications are possible such as decementation of crowns, the development of gingiva inflammation and chipping of the porcelaine layer. The reasons for the complications are patients’ individual physiology, incorrect assessment of the clinical case, violation of clinical and laboratory stages, etc. These reasons include also technological imperfection of manufacturing of dental prosthesis and as a result the adhesion quality of metal frame veneering. Sandblasting is a widespread technology in dental laboratories, but it leads to impurities on the contact surfaces. And the impurities can negatively influence the adhesion. The formation of metal surface morphology and roughness affects adhesion, but nowadays there is no certain way to provide it.

A feature of the plasma-electrolyte treatment is the uniform microdischarges combustion over the entire surface of the crown. With intense combustion melting of the entire structure can occur. To avoid this, regimes were used at lower voltages and currents, which made it possible to implement microlocal melting of the surface without overheating the entire structure. In this regard, the purpose of the study was to determine the optimal parameters of the surface treatment of the metal frame to increase the adhesion of metal and ceramics and to develop a method for forming a microrelief of the surface.

To achieve the result, experimental samples were made of cobalt-chromium alloy. Metal frames were processed by 4 different methods: polishing, milling, sandblasting and plasma-electrolyte treatment. Ceramic layer was applied to the treated surface according to the manufacturer’s recommendations. Morphological and profilometric studies of the samples allowed estimating the dependence between shear strength and the surface roughness. The developed method of microrelief formation by plasma-electrolyte treatment allows obtaining the required surface roughness of metal frame by changing the parameters of gas discharge combustion. At the same time, the geometry of the hollows and protrusions has a spherical shape that can reduce the local stress concentration. It was found that during sandblasting, abrasive sand particles remain on the metal surface and the geometry of the cavities and loads are sharp. According to the results of determining the CVC, a mode (159–178 V, 70–74 °C) was selected to obtain a given value of the roughness parameter Ra 1.45 ± 0.16 µm, which allowed increasing the shear strength of ceramics to the metal samples surfaces up to 8.35 ± 0.21 MPa.

Further research requires studying the effect of plasma-electrolyte microrelief formation on the resulting surface structure in terms of phase composition, microhardness, and Poisson’s ratio. It is very important, since during the combustion of single microdischarges, the point of melting is rapidly cooled in the electrolyte. This cooling leads to an increase in microhardness. And most likely, in the study of microhardness from the surface into the depth of the sample, its gradient decrease will be observed. This will make it possible to create a buffer transition layer from ductile metal to brittle ceramic, thereby reducing the likelihood of ceramic chipping. Therefore, the logical continuation of this work is the study of changes in roughness parameters for different geometries and types of metal frames of dental crowns.

In the same time, the received results make it possible to state an optimistic forecast usage of the plasma-electrolyte processing for improving the adhesion. Of course pull-off strength should be measured for the same roughness values. The evaluation of dependence between normal strength and roughness is planned for future research. Additionally, cyclic tests are mapped out. It is also planned to carry out modeling of a dental prosthesis in the process of chewing by the finite element method.

The proposed method of surfacing allows increasing the shear strength between cobalt-chromium alloy and ceramics. Consequently such an approach increases the vitality of mounted dental construction. The usage of low voltages and currents allows the implementation of the developed method in dental labs and clinics. The plasma-electrolyte processing duration (about 1 min), allows to produce the surfacing during dental’s appointment.

## Figures and Tables

**Table 1:** Firing parameters for ceramic layer.

Mode	T, °C	B, °C	S, min	t, °C/min	H, min	V_1_, °C	V_2_, °C
I	930	403	6	100	2	450	929
II	910	403	4	60	1	450	909
III	900	403	4	60	1	450	899

where T—firing temperature, B—stand-by temperature, S—closing time, t—heating rate, H—holding time, V_1_—vacuum on temperature, V_2_—vacuum off temperature.

**Table 2:** Ra and shear strength results in groups.

Group	Ra, μm	τ, MPa
PL	0.422 ± 0.04	0.26 ± 0.05
MC	0.944 ± 0.11	1.69 ± 0.68
AB50	1.069 ± 0.12	1.05 ± 0.27
AB90	1.243 ± 0.13	3.75 ± 1.24
AB125	2.425 ± 0.26	1.52 ± 0.51
PZ1	1.136 ± 0.15	1.93 ± 0.10
PZ2	1.45 ± 0.16	8.35 ± 0.21
PZ3	1.91 ± 0.21	1.38 ± 0.07

## Data Availability

Not applicable.
